# Supplementary material for: Pregnancy outcomes after snakebite envenomations: A retrospective cohort in the Brazilian Amazonia
Source: PLoS Negl Trop Dis. 2022 Dec 5;16(12):e0010963. doi: 10.1371/journal.pntd.0010963 (PMC9754599; doi:10.1371/journal.pntd.0010963)
Supplement: Attachment — Submitted filename: Rebuttal Letter - Pregnant Women.docx [file pntd.0010963.s003.docx]

**REBUTTAL TO REFEREES**

**PLOS NTD**

**Ms. Ref. No.:** **PNTD-D-22-0105**

**Title:** Pregnancy Outcomes After Snakebite Envenomations: A Retrospective Cohort in the Brazilian Amazonia

**Guest Editor:** Abdulrazaq G. Habib

We sincerely thank the referees for providing important and valuable comments and suggestions, which would help enhance the quality of our article. All the comments are addressed accordingly and have been partially incorporated into the revised manuscript (quoted). Detailed responses to the referee’s concerns and suggestions are described below.

Reviewer #1: Clear objectives, Clear study design.
But info missing on half of the pregnant patients with SBE, see general comments below.

RESPONSE: Changes were made accordingly.

Reviewer #2: Introduction

I am unfamiliar with the meaning of the term ‘anticipation of labor.’ Is this referring to preterm labor? Suggest changing this to more established terminology.
RESPONSE: Change was made accordingly.

Methods

Change ‘one thousand nine-hundred and ninety-six’ to 1,996.
RESPONSE: Change was made accordingly.

The definition of perinatal mortality differs from the WHO definition which is cited in reference 26. I believe the established definition of perinatal mortality is death from 22-weeks gestation until 7 days after birth. I would suggest using a different term (such as ‘combined fetal and neonatal mortality’) or changing the analysis to align with the accepted definition of perinatal mortality.
RESPONSE: The reviewer is completely correct. We fixed the definition in Methods. The analysis was revised, but coincidentally there was no change in the numbers, since our previous definition overlapped with the corrected one for the cases found for this rate.

Reviewer #3: The study is on an important aspect of snakebite, on which there is little information. The objectives are clear and the study population clearly defined. The sample size is adequate, and I have no ethical concerns.
RESPONSE: Thanks for the comment.

However, studies with secondary and retrospective databases have many limitations regarding completeness of information, especially those from remote rural areas in LMIC. This study is no different. Nearly half of the pregnant women with reports of snakebite envenoming during pregnancy had no pregnancy outcome information – this is a major limitation.

RESPONSE: Thanks for the comment. This limitation was discussed in this new version of the manuscript.

--------------------
Reviewer #1: See general comments section

Reviewer #2: My main concern is the large proportion of missing data on pregnancy outcome (around 50%). Pregnancy outcome is the headline finding of the paper and this amount of missing data could have a substantial impact on the result. It seems this was dealt with by listwise deletion and there is no justification as to why this approach was used. Was the data 'missing at random?' If not missing at random, perhaps multiple imputation would have been a better way of dealing with this?
RESPONSE: RESPONSE: The reviewer is completely correct. Unfortunately this was an important limitation when working with these databases. We have added a study limitations paragraph at the end of the Discussion. However, no statistical difference was found between pregnant women with outcome information and missings, in relation to age, ethnicity, literacy, occurrence area (rural or urban), and time to medical assistance after the SBE.

“Like studies with secondary and retrospective databases, this study has limitations related to the quality and completeness of the information. Nearly half of pregnant women with reports of SBEs during pregnancy had no outcome information available in the databases. The possible reasons for this difference are limitations of the surveillance of SBE cases due to errors in self-reporting pregnancy during SBE notification, or due to fetal deaths prior to week 20 of pregnancy, which are not reported in SIM or SINASC. In Brazil, underreporting of live birth records in SINASC has already been verified in the Extra-Amazonian Region of the country, especially in municipalities with lower health infrastructure [51]. Although we do not have estimates of underreporting of live birth records for Amazonia, we believe that these should be higher than in the rest of the national territory, since among traditional populations, especially among indigenous peoples, which are heavily affected by SBEs, deliveries occurring at home are very common [52]. However, the pregnant women with outcome information and missings were similar in relation to sociodemographic variables, suggesting that missing data was not biased.”

Refs:

51. Drumond E de F, Machado CJ, França E. Subnotificação de nascidos vivos: procedimentos de mensuração a partir do Sistema de Informação Hospitalar. Rev. Saude Publica. 2008;42:55–63.

52. Sousa TM, Schweickardt JC, Ferla AA, Lima RTS. A assistência ao parto por parteiras leigas: Uma revisão integrativa. Saúde em Redes. 2022;8:191–205.

Other minor comments:
Change ‘Three thousand two-hundred and ninety-seven’ to 3,297.
RESPONSE: Change was made accordingly.

There is no mention of increased risk of skin necrosis amongst pregnant women in the results section, but this is in the abstract. In table 1 there seems to be no increased risk of skin necrosis between pregnant and non-pregnant women.
RESPONSE: This difference was not really significant. The sentence has been deleted from the Abstract.

Can population incidence of snakebite in pregnancy be calculated? This depends on whether denominator would accurately reflect the total number of pregnancies in the study area during the study period. If this wouldn’t be accurate, then ignore this comment.
RESPONSE: We thank the reviewer for the suggestion. We estimated the rate on 21.7 SBES/100,000 pregnancies (274 cases per 1,263,630 pregnancies in the same period).

Table 5 – could the number of days between the snakebite and the perinatal death be added?
RESPONSE: Table 5 shows the trimester of pregnancy in which the snakebite occurred and gestational week of the perinatal. This was the maximum of precision we could get for this variable.

Reviewer #3: The analysis is appropriate and the data is clearly presented. The Tables are of sufficient clarity.
RESPONSE: Thanks for the comment.
--------------------

Reviewer #1: see general comments section
RESPONSE: Thanks for the comment.

Reviewer #2: It is suggested that placental transfer of venom toxins to the fetus is the most likely explanataion for the higher fetal mortality rate that was observed. The reference (36) for venoms crossing the placenta is a case report of a pregnant woman with snakebite complicated by DIC and placental abruption which resulted in still birth – I cannot see that this demonstrates fetal transfer of toxins (this was fetal death due to abruption). Reference 37 is a general review on drug transfer across the placenta – but not venom toxins. Although evidence is lacking, on balance it would seem unlikely that the large venom toxin proteins would be able to cross the placenta. Damage to the placenta (on the maternal side of the ciculation) seems a more likely mechanism leading to poor fetal outcome.
RESPONSE: Thanks for the observations. All the corrections were made accordingly.

Reference 41 does not seem to be relevant to the statement on fetal coagulopathy (in the case report cited the infant had multiple abnormalities that could have various underlying causes, including microcephaly, hyperbilirubinaemia, anaemia, and thrombocytopaenia. I believe the newborn did not have a coagulopathy).
RESPONSE: We thank the referee for mention that. The correction was made accordingly.

Large proportion of missing data on pregnancy outcome should be highlighted in the discussion with a mention of how this may have affected the results. This could have had a substantial impact on the reported rate of fetal/neonatal death.
RESPONSE: The reviewer is completely correct. Unfortunately this was an important limitation when working with these databases. We have added a study limitations paragraph at the end of the Discussion. However, no statistical difference was found between pregnant women with outcome information and missings, in relation to age, ethnicity, literacy, occurrence area (rural or urban), and time to medical assistance after the SBE.

“Like studies with secondary and retrospective databases, this study has limitations related to the quality and completeness of the information. Nearly half of pregnant women with reports of SBEs during pregnancy had no outcome information available in the databases. The possible reasons for this difference are limitations of the surveillance of SBE cases due to errors in self-reporting pregnancy during SBE notification, or due to fetal deaths prior to week 20 of pregnancy, which are not reported in SIM or SINASC. In Brazil, underreporting of live birth records in SINASC has already been verified in the Extra-Amazonian Region of the country, especially in municipalities with lower health infrastructure [51]. Although we do not have estimates of underreporting of live birth records for Amazonia, we believe that these should be higher than in the rest of the national territory, since among traditional populations, especially among indigenous peoples, which are heavily affected by SBEs, deliveries occurring at home are very common [52]. However, the pregnant women with outcome information and missings were similar in relation to sociodemographic variables, suggesting that missing data was not biased.”

Refs:

51. Drumond E de F, Machado CJ, França E. Subnotificação de nascidos vivos: procedimentos de mensuração a partir do Sistema de Informação Hospitalar. Rev. Saude Publica. 2008;42:55–63.

52. Sousa TM, Schweickardt JC, Ferla AA, Lima RTS. A assistência ao parto por parteiras leigas: Uma revisão integrativa. Saúde em Redes. 2022;8:191–205.

Reviewer #3: It may be difficult to arrive at firm conclusions with a significant limitation of information regarding pregnancy outcomes in snakebite victims (nearly half). The limitations should be discussed in more detail and not limited to a short paragraph at the end of the discussion.

RESPONSE: The reviewer is completely correct. Unfortunately this was an important limitation when working with these databases. We have added a study limitations paragraph at the end of the Discussion. However, no statistical difference was found between pregnant women with outcome information and missings, in relation to age, ethnicity, literacy, occurrence area (rural or urban), and time to medical assistance after the SBE.

“Like studies with secondary and retrospective databases, this study has limitations related to the quality and completeness of the information. Nearly half of pregnant women with reports of SBEs during pregnancy had no outcome information available in the databases. The possible reasons for this difference are limitations of the surveillance of SBE cases due to errors in self-reporting pregnancy during SBE notification, or due to fetal deaths prior to week 20 of pregnancy, which are not reported in SIM or SINASC. In Brazil, underreporting of live birth records in SINASC has already been verified in the Extra-Amazonian Region of the country, especially in municipalities with lower health infrastructure [51]. Although we do not have estimates of underreporting of live birth records for Amazonia, we believe that these should be higher than in the rest of the national territory, since among traditional populations, especially among indigenous peoples, which are heavily affected by SBEs, deliveries occurring at home are very common [52]. However, the pregnant women with outcome information and missings were similar in relation to sociodemographic variables, suggesting that missing data was not biased.”

Refs:

51. Drumond E de F, Machado CJ, França E. Subnotificação de nascidos vivos: procedimentos de mensuração a partir do Sistema de Informação Hospitalar. Rev. Saude Publica. 2008;42:55–63.

52. Sousa TM, Schweickardt JC, Ferla AA, Lima RTS. A assistência ao parto por parteiras leigas: Uma revisão integrativa. Saúde em Redes. 2022;8:191–205.

--------------------
Use  this section for editorial suggestions as well as relatively minor modifications of existing data that would enhance clarity. If the only modifications needed are minor and/or editorial, you may wish to recommend “Minor Revision” or “Accept”.

Reviewer #1: see general comments
RESPONSE: Thanks for the comments.

Reviewer #2: (No Response)
RESPONSE: Thanks for the comments.

Reviewer #3: There are several minor typographical errors, but overall the paper is well written, and I enjoyed reading it.

RESPONSE: Thanks for the comments. An English-speaking reviewer corrected the manuscript.

--------------------
Reviewer #1: The manuscript discusses pregnancy & snakebite envenoming which is highly needed data. There are nevertheless two major challenges I see based on the manuscript. For half of the pregnant women with a snakebite envenoming there were no pregnancy outcomes available. The authors repot one of the reasons of unknown outcomes is fetal deaths before week 20 which are not included in the used databases. That suggests that there may be a selection included in the manuscript not reflecting the overall outcomes. The residence of the victims is known; is it with further ethical approval possible to obtain the hospital data from these missing patients? Even if the outcomes of half of these missings can be obtained, that would be useful.

RESPONSE: The reviewer is completely correct. Unfortunately this was an important limitation when working with these databases. We have added a study limitations paragraph at the end of the Discussion. However, no statistical difference was found between pregnant women with outcome information and missings, in relation to age, ethnicity, literacy, occurrence area (rural or urban), and time to medical assistance after the SBE.

“Like studies with secondary and retrospective databases, this study has limitations related to the quality and completeness of the information. Nearly half of pregnant women with reports of SBEs during pregnancy had no outcome information available in the databases. The possible reasons for this difference are limitations of the surveillance of SBE cases due to errors in self-reporting pregnancy during SBE notification, or due to fetal deaths prior to week 20 of pregnancy, which are not reported in SIM or SINASC. In Brazil, underreporting of live birth records in SINASC has already been verified in the Extra-Amazonian Region of the country, especially in municipalities with lower health infrastructure [51]. Although we do not have estimates of underreporting of live birth records for Amazonia, we believe that these should be higher than in the rest of the national territory, since among traditional populations, especially among indigenous peoples, which are heavily affected by SBEs, deliveries occurring at home are very common [52]. However, the pregnant women with outcome information and missings were similar in relation to sociodemographic variables, suggesting that missing data was not biased.”

Refs:

51. Drumond E de F, Machado CJ, França E. Subnotificação de nascidos vivos: procedimentos de mensuração a partir do Sistema de Informação Hospitalar. Rev. Saude Publica. 2008;42:55–63.

52. Sousa TM, Schweickardt JC, Ferla AA, Lima RTS. A assistência ao parto por parteiras leigas: Uma revisão integrativa. Saúde em Redes. 2022;8:191–205.

Authors also describe the antivenom currently used is not crossing the placenta. But the study described a 15 years period; do the authors know what is used earlier? And if not, how do we than know if the pregnancy outcomes are related to the snakebite envenoming instead of the antivenom/allergic reactions?
RESPONSE: For more than a century (since 1901), the antivenoms in Brazil are consisted of horse derived antibodies. In order to overcome the hypersensitivity reactions caused by the horse-derived antibodies, many antivenom manufacturers (including Brazilian Institute) digest the whole IgG using pepsin to produce bivalent F(ab')2 fragments, which lack the C terminal Fc domains. Indeed, the digestion is related to reduce complex-mediated or type III hypersensitivity reactions, albeit the molecule retains divalency and the ability to bind complement. In general, adverse reactions to Brazilian antivenoms are not very frequent (~10%) and are limited to urticaria and pruritus (cases of anaphylaxis are very rare) (1,2). Thus, we do not believe that reactions to antivenoms could be associated with the poor pregnancy outcomes found in this study.

1. Soares FGS, Ibiapina HN, Sartim MA, Mendonça-da-Silva I, Nascimento EF, Ferreira LCL, Cerni FA, Malheiro A, Pucca MB, Wen FH, Maria Moura-da-Silva A, Costa AG, Monteiro WM, Sachett JAG. Lower levels of CXCL-8 and IL-2 on admission as predictors of early adverse reactions to Bothrops antivenom in the Brazilian Amazon. Cytokine. 2022 Apr;152:155825.

2. Mendonça-da-Silva I, Magela Tavares A, Sachett J, Sardinha JF, Zaparolli L, Gomes Santos MF, Lacerda M, Monteiro WM. Safety and efficacy of a freeze-dried trivalent antivenom for snakebites in the Brazilian Amazon: An open randomized controlled phase IIb clinical trial. PLoS Negl Trop Dis. 2017 Nov 27;11(11):e0006068.

Intro:
Line 96: You describe the clinical presentation of Bothrops bites but there is no info how common Bothrops bites are in Brazil. This follows in line 151. Suggest to combine that background. It deserves a place in the introduction and not only in the methods.
RESPONSE: Thanks for the observation. The info was added in the introduction, see lines 85-86.

Line 99-100: can you check this sentence? It’s a bit hard to read.
RESPONSE: The whole paragraph was reviewed. See lines 97-106.

Line 133: Can you provide more info on the databases used? How many persons are in the database? And what about privacy? And data management? Who did the blocking? In how many pregnant women were you not able to find a match apart from the pregnancy outcomes?
RESPONSE: Databases used are all described in the Methods in this new version of the manuscript and the numbers are shown in the flowchart in Figure 1.

Line 158-170: variables collected but not clear yet how these relate to pregnancy and SBE, eg why is data on race/ethnicity collected?
RESPONSE: Sociodemographic variables are known to be related to snakebite outcomes, including ethnicity. The aim of presenting these variables in this study is to describe women of childbearing age and especially pregnant women affected by snakebites.

The data are from 2007-2021; any changes in that period when thinking about pregnancy outcomes and the Zika time? Apart from zika, how does are the negative outcomes placed in time? Are they more often > 10 years ago or distributed over time evenly?
RESPONSE: The reviewer's doubt is pertinent. Unfortunately, we did not use the Zika virus nominal bank to verify a potential bias of Zika virus infection in our results. Analyzing the temporal distribution of the poor outcomes identified in this study, however, there is no concentration in the years with the highest incidence of Zika. In addition, we do not see plausibility in the greater burden of Zika among groups of pregnant women with and without SBEs.

Line 207: why does gender need to be included here?

RESPONSE: Thanks for the comment. Reviewer is correct. We deleted gender.

Line 246: instead of a p-value, a difference in age with CI would be more informative here.

RESPONSE: Change was made accordingly.

Line 253: How was the diagnosis Bothrops made? And can you add info in the introduction on the number of dry bites in Bothrops bites?
RESPONSE: In Brazil, snakebite diagnosis is made based in clinico-epidemiological information from the patients, according the MoH guidelines. We included the proportion of Bothrops cases in the Introduction.

Line 254-255: how do these percentages relate to percentages in child bearing age/not pregnant?  How is unclottable blood defined?
RESPONSE: The percentages in both groups are presented in Figure 1 and Table 1. Unclottable blood in Brazil is determined by the Lee-White clotting test (information added to Methods).

Line 263: remove p value, keep OR and CI, same for OR’s in rest of manuscript. Once we have the OR and CI the pvalue is of no added benefit.

RESPONSE: Change was made accordingly.

Line 266: since numbers are so low, include absolute values apart from percentages.

RESPONSE: Numbers were added accordingly.

Line 287: remind us how many have missing outcomes and include in discussion (see first lines of review)

RESPONSE: The reviewer is completely correct. Unfortunately this was an important limitation when working with these databases. We have added a study limitations paragraph at the end of the Discussion. However, no statistical difference was found between pregnant women with outcome information and missings, in relation to age, ethnicity, literacy, occurrence area (rural or urban), and time to medical assistance after the SBE.

“Like studies with secondary and retrospective databases, this study has limitations related to the quality and completeness of the information. Nearly half of pregnant women with reports of SBEs during pregnancy had no outcome information available in the databases. The possible reasons for this difference are limitations of the surveillance of SBE cases due to errors in self-reporting pregnancy during SBE notification, or due to fetal deaths prior to week 20 of pregnancy, which are not reported in SIM or SINASC. In Brazil, underreporting of live birth records in SINASC has already been verified in the Extra-Amazonian Region of the country, especially in municipalities with lower health infrastructure [51]. Although we do not have estimates of underreporting of live birth records for Amazonia, we believe that these should be higher than in the rest of the national territory, since among traditional populations, especially among indigenous peoples, which are heavily affected by SBEs, deliveries occurring at home are very common [52]. However, the pregnant women with outcome information and missings were similar in relation to sociodemographic variables, suggesting that missing data was not biased.”

Refs:

51. Drumond E de F, Machado CJ, França E. Subnotificação de nascidos vivos: procedimentos de mensuração a partir do Sistema de Informação Hospitalar. Rev. Saude Publica. 2008;42:55–63.

52. Sousa TM, Schweickardt JC, Ferla AA, Lima RTS. A assistência ao parto por parteiras leigas: Uma revisão integrativa. Saúde em Redes. 2022;8:191–205.

293: deaths before week 20 would be crucial info to get.

RESPONSE: Unfortunately, fetal deaths prior to week 20 of pregnancy are not reported in SIM or SINASC, or other databases, in Brazil.

302/303: how many of the patients with poor outcomes did receive antivenom? Include in results in summarized way.

RESPONSE: We added the information about the use of antivenoms in pregnant women in which perinatal deaths was the outcome.

307: talks about 3 deaths, but in line 309 and 312 talks about four cases.

RESPONSE: Actually, there are four cases. Sentence was re-written accordingly.

Line 350: provide CI of the 4%.
RESPONSE: This rate comes from the cited reference, which he did not present CI 95%.

Reviewer #2: Overall I thought this was an excellent study and provides a rare insight into pregnancy outcome in snakebite. The large samples size and representative population are strengths. The main limitation is the large proportion of missing on pregnancy outcomes. I feel there needs to be more careful consideration of how best to deal with this missing data.
RESPONSE: The reviewer is completely correct. Unfortunately this was an important limitation when working with these databases. We have added a study limitations paragraph at the end of the Discussion.

Reviewer #3: The paper discusses a neglected area of snakebite, where there are misconceptions on the management even among physicians (eg. use of antivenom). It is clearly and important topic. The major limitation is use of secondary data which the authors themselves admit may be incomplete and unreliable in the study setting. The results clearly show this, with a significant gap in data on pregnancy outcome - an important objective of the study. The authors should to describe work in this area from other parts of the world, even though they are small studies.

RESPONSE: The reviewer is completely correct. There is a scarcity of literature on the topic around the world. We have tried to make a good review of these references in this new version of the manuscript.
