# Supplementary material for: Pregnancy outcomes after snakebite envenomations: A retrospective cohort in the Brazilian Amazonia
Source: PLoS Negl Trop Dis. 2022 Dec 5;16(12):e0010963. doi: 10.1371/journal.pntd.0010963 (PMC9754599; doi:10.1371/journal.pntd.0010963)
Supplement: Attachment — Submitted filename: Rebuttal Letter - Pregnant Women 2.docx [file pntd.0010963.s004.docx]

**REBUTTAL TO REFEREES**

**PLOS NTD**

**Ms. Ref. No.:** **PNTD-D-22-0105**

**Title:** Pregnancy Outcomes After Snakebite Envenomations: A Retrospective Cohort in the Brazilian Amazonia

**Guest Editor:** Abdulrazaq G. Habib

We sincerely thank the referees for providing important and valuable comments and suggestions, which would help enhance the quality of our article. All the comments are addressed accordingly and have been partially incorporated into the revised manuscript (quoted). Detailed responses to the referee’s concerns and suggestions are described below.

Reviewer #1: As a reviewer I am not convinced by the data though I understand it's been a lot of work to get this data, the authors do not explain why it is not possible to try and get the other 50% of the data on outcomes. They do explain that the characteristics of the pregnancies in SBE victims were similar to the other 50% of the data, but that is not my main worry.
There were 4 neonatal deaths in the current group. Only 4 deaths to base the Odds ratios on. My problem is that such a low number of deaths may be influenced both by chance or selection (not based on characteristics of the mother). Sick mothers may present to the hospital -> included in the numbers, whereas the home births may actually have lower deaths. Or even by chance, the number of deaths in the other half of the population under study may have been between 0 and 6 which would have influenced OR heavily.
Furthermore, pregnancy outcomes < 20 weeks are not available. Whereas almost all women with fetal death or neonatal death were bitten in the first trimester... How can the authors explain that they link the neonatal death with a bacterial sepsis after birth to the snakebite in the first trimester. It is biologically more difficult to explain than having a -by chance finding- or a  selection bias here; the number of deaths higher in the counted SBE victims than in the non-counted SBE victims (who did not present to the hospital).

RESPONSE:

We thank the reviewer for the comment and point out that we share the same concern. We really thought about how to collect the missing data, from the first phase of the study, but realized that the task would be practically impossible, for a few reasons: 1) First, the databases rarely have the pregnant women's address and phone fields filled in. We believe that most should not even have a phone. As for addresses, they live in remote communities on the banks of rivers or in indigenous villages, and do not have a regular address as we know it (with street, number, zip code). The few phones informed have no answered calls. We know from other projects that the exchange of numbers is very common in the Amazonian population. We were not successful in this strategy.

Another way would be to access information from medical records in health units. However, as we highlighted in the first review, most of them must have given birth at home, and do not have hospital records. We remind you that because these pregnant women are only in the SINAN database (snakebite report), but not in the SINASC (data on pregnancy outcome), the only health unit that we could look for information is the unit that treated snakebite, not the unit that provided obstetric care. Another important factor is that in some cases where we have information from the unit that could inform the outcome of the pregnancy, the units reported that they did not even have medical records stored (they were discarded after a short time due to lack of space), or, if they did, they were all paper medical records, and they had no way of responding to our request due to the lack of a person to carry out the search. We remind you that the state has at least 80 units with obstetric service.

So, the number of cases cannot change, unfortunately. Likewise, about pregnancy outcomes <20 weeks are not available, as already highlighted in the manuscript and first Rebuttal Letter, it is not recorded in any database.

We understand that this is a major limitation of the study, but we also emphasize that hardly another LMIC in the world, endemic for snakebites, would have the data that we used here, even with so many limitations. This is a preliminary study, the first of its kind, which generated important hypotheses, which may or may not be confirmed in the future, in cohorts of pregnant women followed up in a planned manner. Therefore, we respectfully request the reviewer to consider these explanations when making his decision.

About the “link between neonatal death with a bacterial sepsis after birth to the snakebite in the first trimester”, we explain as follows:

Bacterial sepsis appeared in 3 of the 8 perinatal deaths, as one of the causes, but not the only cause reported on death certificates (Table 5). Unfortunately, there is no way to test in this work whether there is a direct relationship between snakebite in the first trimester of pregnancy and neonatal sepsis. Nevertheless, we do not consider this relationship to be implausible: as we explained in the discussion, snakebites could result in placental dysfunction and intrauterine growth restriction, known risk factors for neonatal sepsis. Placental dysfunction could decrease the amount of blood and nutrition supply leading to intrauterine growth retardation and increased risk of neonatal complications leading to increased risk of neonatal infection and sepsis (1). Likewise, the impaired intrauterine development of immunological system in preterm births is considered the leading factor for increased neonatal susceptibility to sepsis. The reduced immunological competence, represented by immature function of blood leukocytes and reduced immunoglobulin (due to maternal transference to fetus) contributes to a innacurate immune response against infectious agents, making premature infants at much higher risk for sepsis when compared to term infants (2).

1. Lamont R. F. The role of infection in preterm labour and birth. *Hospital Medicine* . 2003;64(11):644–647.

2. Raymond SL, Stortz JA, Mira JC, Larson SD, Wynn JL, Moldawer LL. Immunological Defects in Neonatal Sepsis and Potential Therapeutic Approaches. Front Pediatr. 2017;5:14.

We included this major limitation also in the Abstract.

--------------------

Reviewer #3: In the revised version of the paper, the authors have expanded the paragraph on limitations of the study. However, given that losing 50% of data is a major problem as commented on by all three reviewers, they must emphasize this limitation more. They should state that this is a MAJOR limitation, and state that it may be difficult to arrive at firm conclusions with lack of information regarding pregnancy outcomes in nearly half the snakebite victims.

RESPONSE: In this new version, we state that this is a MAJOR limitation.

--------------------

Reviewer #3: The authors must emphasize the limitation of losing information on nearly half the pregnant women more. They should state that this is a MAJOR limitation. See comment in section on Conclusions.
RESPONSE: In this new version, we state that this is a MAJOR limitation.
--------------------
